# Socioeconomic factors outweigh perceived built environmental influences on self-rated health among middle-aged and older adults in western China

**DOI:** 10.3389/fsoc.2026.1784715

**Published:** 2026-03-24

**Authors:** Lansicheng Yao, Jacquline Tham, Qing Pan, Xiaobing Tian

**Affiliations:** 1 Foreign Affairs Office, North Sichuan Medical College, Nanchong, Sichuan, China; 2 Graduate School of Management, Postgraduate Center, Management and Science University, Shah Alam, Selangor, Malaysia; 3 Health Management Center, General Practice Medical Center, Innovation Institute for Integration of Medicine and Engineering, West China Hospital, Sichuan University, Chengdu, China; 4 School of Public Health, North Sichuan Medical College, Nanchong, Sichuan, China

**Keywords:** perceived built environment, self-rated health, socioeconomic factors, physical activity, body mass index

## Abstract

**Objective:**

Previous environmental evidence has demonstrated that supportive environments can improve population health by reducing environmental hazards. However, the extent to which such environments can further enhance health outcomes by promoting physical activity and encouraging proactive health behaviors remains unclear once socioeconomic inequalities are considered.

**Method:**

A cross-sectional community survey was conducted among community-dwelling middle-aged and older residents in Nanchong, China. The validated Chinese version of the Neighborhood Environment Walkability Scale–Abbreviated (NEWS-A), the SF-36 General Health subscale, and the Chinese short form of the International Physical Activity Questionnaire (IPAQ-SF) were used to measure perceived built environment, self-rated health and physical activity, respectively. A structural equation model (SEM) was employed to test whether the perceived built environment was associated with self-rated health via physical activity and body mass index. Model estimation utilized the WLSMV method to handle ordinal data, with model fit rigorously assessed using standard indices and a prior confirmatory factor analysis. Direct, indirect, and total effects were estimated with robust standard errors.

**Results:**

Among 3,753 participants (mean age 50.1 years; 39.5% men), the final SEM showed excellent fit. After adjustment for socioeconomic and demographic covariates, perceived built environment did not affect self-rated health (all *p* > 0.05), and neither physical activity (β = −0.038, *p* = 0.180) nor body mass index (β = 0.028, *p* = 0.319) mediated the relationship. Self-rated health was primarily influenced by household income (β = 0.369, *p* < 0.001), age (β = 0.016, *p* < 0.001), education (β = −0.121, *p*= 0.004), and urban residency (β = 0.135, *p* = 0.006).

**Conclusion:**

After controlling socioeconomic and demographic characteristics, the perceived built environment showed no independent association with self-rated health. This suggests that social and economic gaps may outweigh and potentially obscure the health benefits of supportive neighborhood environments in similar contexts.

## Graphical Abstract

**Figure:**
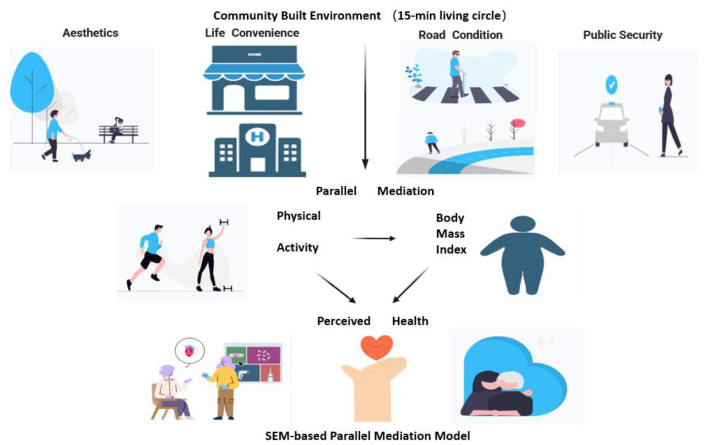

## 1 Introduction

Self-rated health (SRH) is a widely used holistic indicator of physical, psychological, and social wellbeing and is strongly associated with morbidity, functional decline, and all-cause mortality in later life (WHO, 2021; Basumatary et al., 2024). Large-scale studies among older adults consistently show that those reporting poorer SRH have higher risks of hospitalization, disability, and deat, and More than one-third of older adults in certain countries assess their health as “poor” or “fair,” highlighting a substantial burden of perceived ill health in aging societies (Chen et al., 2023; OECD, 2023). Therefore, strategies targeting modifiable determinants of SRH are important topics in both clinical practice and public health.

Perceived built environments have been proposed as one such determinant (Mu et al., 2024). Supportive environments, characterized by convenient access to daily facilities, walkable streets, favorable traffic conditions, and good public safety, have been linked to higher physical activity and, in some studies, to better self-rated health among older adults (DeSalvo et al., 2006; Jylhä, 2009). However, empirical findings are heterogeneous across settings. While some studies identify consistent positive effects of specific dimensions, such as daily facilities and green walkable stress, others report null or divergent associations based on neighborhood design and measurement methods (Wang and Zhao, 2012; Wang et al., 2025). Much of the existing literature is rooted in developed-country contexts, where neighborhood planning expressly emphasizes public physical activity (PA) facilities to promote exercise (Kong et al., 2025; Li and Shen, 2025).

Furthermore, perceived built environmental features are highly relevant to socioeconomic and demographic conditions (Gao et al., 2024). Socioeconomic status, including education, income, and place of residency, exerts a profound and well-documented influence on health outcomes (Fan et al., 2023; Li et al., 2024; Chen et al., 2025). Recent cohort and review studies report clear social gradients, meaning older adults with lower incomes or living in rural or deprived areas are substantially more likely to report poor SRH than their more advantaged peers (Abdi et al., 2019; Salminen et al., 2025). Although socioeconomic inequalities are highly correlated with the distribution of community infrastructure, it remains insufficiently understood how these fundamental socioeconomic factors might outweigh perceived built environmental factors in shaping the self-rated health among community-dwelling middle-aged to older adults (Robles et al., 2023; Tangang et al., 2026).

These issues are highly relevant in western China, where rapid population aging coincides with uneven urban-rural development. In Sichuan Province, the proportion of residents aged ≥60 years already exceeds the national average (nationally 15.4% in 2024; Sichuan: 14%−20%) (Google Scholar, 2024; Zhou, 2024). In Nanchong City, 26% of its 5.6 million residents are aged 60 or older (Honghei Statistical Bulletin Database, 2020).

Notably, the conceptualization of the perceived built environment in China differs fundamentally from developed countries' paradigms. In western China, rural areas are dominated by self-built, independent housing, while urban areas consist of high-density, gated communities (Yip et al., 2019). For these residents, a “supportive” built environment is primarily defined by the convenience of transportation, daily facilities, and access to healthcare, rather than by the availability of dedicated physical activity (PA) facilities (Zeng et al., 2026; Chen et al., 2024). Consequently, the traditional developed countries' assumption that perceived built environments improve health outcomes primarily by promoting physical activity may not hold in this context.

This study addresses this gap by analyzing community-dwelling middle-aged and older adults in Gaoping District, Nanchong. It aimed to: (1) examine associations between perceived built environment and SRH; (2) assess whether PA and BMI mediate these associations; and (3) compare the strength of associations between socioeconomic and demographic characteristics and SRH with those of perceived built environment factors.

## 2 Methods

### 2.1 Study design and participants

A cross-sectional study was conducted in Nanchong, Sichuan Province, between July 2023 and August 2024. Ethical approval was obtained from the Institutional Review Boards of the participating universities (Approval No. 2024019). Written informed consent was obtained from all participants before data collection.

A multi-stage cluster random sampling approach was used. Three townships and two sub-districts were randomly selected, and within them, village or residents' committees were chosen; neighboring committees were included when necessary. From each selected committee, 164 households were randomly sampled, yielding a total of 4,100 households. Within each household, one permanent resident aged 18 years or older was selected using the nearest-birthday method. The present analysis was restricted to community-dwelling adults aged ≥45 years, resulting in a final sample of 3,753 participants.

A multi-stage cluster random sampling approach was employed to select study participants. In detail, three townships and two sub-districts were randomly selected, among which five village committees were randomly chosen (Supplementary Table S2); neighboring committees were included when necessary. From each selected committee, 164 households were randomly sampled, yielding a total of 4,100 households. Within each household, one permanent resident aged 18 years or older was selected using the nearest-birthday method. The final valid sample comprised 3,753 participants. The sample size was calculated based on the formula: $n=\frac{Z_{1-\alpha /2}^{2}p\left(1-p\right)}{d^{2}}$.

Data for this study were derived from a comprehensive community health survey in Nanchong. To ensure sufficient statistical power for capturing various chronic conditions in the parent survey. Sample size was calculated using the single proportion formula with a 95% confidence level (*Z* = 1.96), expected prevalence of 10.3% (diabetes, a representative chronic disease in Sichuan Province, 2013), design effect = 2, and allowable relative error of 23%. This yielded a minimum of 1,266 per stratum. After adjusting for gender stratification and 5% non-response, the final target was 2,700; the sample size achieved was 3,753.

### 2.2 Participants

Participants were invited through community general practitioners and completed structured questionnaires under investigator supervision. The survey followed a fixed order: the modified NEWS-A was assessed perceived built environment, followed by the Chinese IPAQ-SF to record physical activity over the preceding 7 days; and ultimately, the SF-36 general health subscale to evaluate SRH.

Inclusion criteria were: (1) aged 45 years or older; (2) residing in the community under local management for at least 6 months; (3) able to complete the questionnaire independently or with assistance; and (4) provision of written informed consent. Exclusion criteria were: (1) had no history of psychiatric disorders; (2) acute illness during the survey; and (3) inability to communicate effectively.

### 2.3 Study procedure and data collection

All interviewers received standardized training on survey protocols, informed consent, and neutral interviewing techniques. For participants with low literacy, visual impairment, or limited mobility, questions were read aloud, and responses were recorded verbatim in face-to-face interviews. Approximately 10% of interviews were conducted at participants' homes when they were unable to attend the survey site.

Anthropometric measurements, including height and weight, were obtained using calibrated instruments according to WHO protocols; body mass index (BMI) was calculated as weight (kg)/height (m^2^). Data were double-entered independently by two clerks using Excel, reconciled via range and logic checks, and subsequently exported to SPSS 27.0 (IBM Corp, Armonk, NY, United States) and R (version 4.x) for analysis. Quality control comprised random back-checks of 5% of households, time-stamp checks, and verification of community codes.

### 2.4 Measurements

#### 2.4.1 Socio-demographic characteristics and related variables

Socio-demographic characteristics, including age, gender, marital status, education level, area of residence, height, weight, household income, and information on family members and carers, were collected by trained interviewers using a structured questionnaire. Family economic status was classified as low, medium, or high based on the participants' self-assessment. Potential covariates were collected during the survey; variable definitions and coding are provided in Supplementary Table S1.

#### 2.4.2 Perceived built environment

The perceived built environment was assessed using the validated Chinese version of the Neighborhood Environment Walkability Scale–Abbreviated (NEWS-A) (Jia et al., 2014; Bailey-Catalán et al., 2019). It comprises 17 items categorized into five dimensions: convenience of supporting living facilities, road conditions, aesthetics, traffic conditions, and public safety. Responses are coded on a 5-point Likert scale, where 1 indicates strong disagreement (very bad), and 5 indicates strong agreement (very good); higher scores reflect more supportive perceived built environments. In the present analysis, the NEWS-A scale was used to capture residents' perceptions of their immediate neighborhood context, which is conceptually consistent with self-rated health as a subjective health outcome and has been widely applied in community-based studies of Chinese adults and older people (Cerin et al., 2010).

#### 2.4.3 Self-rated health

Self-rated health was assessed by the General Health (GH) subscale of the Chinese version of the 36-Item Short Form Health Survey (SF-36), validated in Chinese populations (Shen et al., 2014; Ware and Sherbourne, 1992). The GH subscale contains five items rated on a 5-point Likert scale. Items were recorded so that higher scores consistently reflect better health and then averaged to yield a composite GH score ranging from 1 to 5 (higher = better perceived health). This 1–5 metric was used in all descriptive statistics and SEM analyses.

#### 2.4.4 Physical activity

Physical activity (PA) was evaluated utilizing the Chinese short form of the International Physical Activity Questionnaire (IPAQ-SF) (Craig et al., 2003; Jia et al., 2014; Hu et al., 2015), which has shown good reliability and validity in Chinese adults for measuring weekly walking, moderate, and vigorous activities (Craig et al., 2003; Cerin et al., 2010; Hu et al., 2015). To reduce artifacts from extreme reporting, analyses were capped at 180. Due to the significant right skew of total MET, we implemented a natural-log transformation [ln (MET+1)] for modeling, while raw values were reported for descriptive analyses. Participants were classified into low, moderate, or high physical-activity groups for descriptive purposes only.

### 2.5 Statistical analysis

#### 2.5.1 Methods

##### 2.5.1.1 Software

All statistical analyses were performed in R (R Foundation for Statistical Computing, Vienna, Austria). The primary packages used were tidyverse (data management), gtsummary (descriptive statistics), Hmisc (correlation analyses), EFA tools and psych [Kaise-Meyer-Olkin (KMO), Barlett's test, and exploratory factor analysis (EFA)], lavaan [structural equation modeling (SEM) estimation using WLSMV and theta parameterization], and pheatmap (heat-map visualization). Additional packages loaded in the environment were not utilized in the main SEM analysis.

##### 2.5.1.2 Data preparation and variable construction

IPQA-SF-based metabolic equivalent (MET) minutes were computed following the official scoring protocol. For vigorous, moderate, and walking activities, total daily minutes were first obtained as (hours × 60) + minutes, and total weekly minutes were calculated as (frequency × daily minutes). Weekly MET-minutes were then derived using the coefficients 8.0, 4.0, and 3.3 for vigorous, moderate, and walking activity, respectively. The total weekly PA (Total MET, MET-min/week) and BMI were derived as described in Section 2.4. Observations with extreme Total MET values (>3,000 MET-min/week) were excluded, and all continuous variables were inspected for outliers and distributional anomalies before analysis.

##### 2.5.1.3 Descriptive analyses

Descriptive statistics were generated for all study variables. Continuous variables were summarized as mean ± standard deviation, and categorical variables were summarized as number (percentage). The distributions of BMI and Total MET were examined visually using histograms to assess normality and the presence of extreme values (see Supplementary Figures S1, S2).

##### 2.5.1.4 Correlation analyses

Pairwise Spearman and Kendall correlation coefficients were computed to estimate the strength and direction of associations between the seventeen community-built environment variables (Com.Env1–Com.Env17), physical activity (Total MET), BMI, and self-rated health (SF.Total). Correlation matrices were visualized using heat maps to illustrate the overall pattern of associations (shown in Supplementary Figures S3, S4).

##### 2.5.1.5 Assessment of factorability and distributional assumptions

The suitability of perceived built environment items (Com.Env1–Com.Env17) for factor analysis and structural equation modeling (SEM) was assessed using the Kaiser–Meyer–Olkin (KMO) measure of sampling adequacy and Bartlett's test of sphericity. Shapiro–Wilk tests were applied to evaluate univariate normality, and Mardia's test was employed to examine multivariate skewness and kurtosis. Because both univariate and multivariate normality assumptions were violated, robust estimation procedures for ordinal, non-normally distributed data were applied in subsequent analyses.

##### 2.5.1.6 Confirmatory factor analysis (CFA)

A confirmatory factor analysis (CFA) was conducted on Com.Env1–Com.Env17 to verify the latent structure of the perceived built environment construct. The minimum residual (minres) extraction method with oblimin (oblique) rotation was applied, and the number of factors was fixed *a priori* at five, consistent with the theoretical framework of NEWS-A. Factor loadings of ≥0.40 were considered significant. Standard summary statistics, including the sum of squared loadings, proportion variance, and cumulative variance explained, were reported.

#### 2.5.2 Structural equation modeling (SEM)

##### 2.5.2.1 Effect decomposition

Direct, indirect, and total effects were computed based on the parameter labels defined in the model. Robust standard errors, *z*-statistics, and 95% confidence intervals were obtained using standardized estimates from lavaan. The indirect and total effects were calculated as follows:


$$\begin{array}{l} \text{Indirect\_}\left(\text{DA}\to \text{OB}\to \text{SRH}\right)=\text{b}\times \text{c},\text{ Total\_}\left(\text{DA}\to \text{SRH}\right)\\ \;=\text{d}+\text{b}\times \text{c}. \end{array}$$

For each community environment factor k ε {1,3,5}, the single and double mediation effects were defined as:


$$\begin{array}{l} \text{Indirect\_}\left(\text{CE\_k}\to \text{DA}\to \text{SRH}\right)=\text{a\_k}\times \text{d},\text{ Indirect\_}(\text{CE\_k}\\ \to \text{DA}\to \text{OB}\to \text{SRH})=\text{a\_k}\times \text{b}\times \text{c},\text{ Total\_}(\text{CE\_k}\\ \to \text{SRH})=\text{a\_k d}+\text{a\_k b c}. \end{array}$$

Direct paths from CE factors to SRH were not specified; therefore, total effects were equivalent to the sum of indirect effects. The total effects of CE1, CE3, and CE5 on obesity through physical activity were calculated as a_k × b. All tests were two-tailed, and a significance level of 0.05 was adopted.

## 3 Results

### 3.1 Description of included participants

A total of 3,753 participants were included in the study (mean age: 50.1 ± 17.2 years; 39.5% male). The mean self-rated health score was 2.91 ± 1.23 (Table 1). Most participants were of Han ethnicity (99.3%), 75.6% were married, and 15.4% had attained a bachelor's degree or higher. Mean height, weight, and waist circumference were 159.5 ± 8.6 cm, 64.8 ± 19.7 kg, and 77.9 ± 18.5 cm, respectively; mean systolic blood pressure was 120.4 ± 24.3 mmHg. Regarding residence, 43.4% lived in urban areas, 29.7% in towns, and 27.0% in rural areas. Family economic status was predominantly “average” (67.5%). Overall, 26.0% of participants were classified as having high PA.

**Table 1:** Baseline characteristics of the study population (*N* = 3,753).

**Characteristic**	**Overall (*N* = 3,753)**
**Gender (*****n*** **%)**	
Male	1,481 (39.5)
Female	2,272 (60.5)
Age (years)	50.13 ± 17.23
**Ethnicity (*****n*** **%)**	
Han	3,726 (99.3)
Others	27 (0.7)
**Marriage (*****n*** **%)**	
Single	490 (13.1)
Married	2,837 (75.6)
Widows	314 (8.4)
Divorced	112 (3.0)
**Education (*****n*** **%)**	
No formal education	468 (12.5)
Elementary school or below	794 (21.2)
Middle school	608 (16.2)
High school/vocational school/technical school	1,304 (34.7)
Bachelor's degree or above	579 (15.4)
**Residency (*****n*** **%)**	
Rural	1,012 (27.0)
Town	1,114 (29.7)
Urban	1,627 (43.4)
**Family economics (*****n*** **%)**	
Very good	143 (3.8)
Good	449 (12.0)
Average	2,532 (67.5)
Poor	514 (13.7)
Very poor	115 (3.1)
**Anthropometrics**	
Height (cm)	159.48 ± 8.61
Weight (kg)	64.77 ± 19.65
Waist (cm)	77.88 ± 18.49
SBP (mmHg)	120.41 ± 24.30
**Physical activity (*****n*** **%)**	
High PA (yes/no)	727 (26.0)/2,066 (74.0)
Medium PA (yes/no)	1,535 (55.0)/1,258 (45.0)
Walk PA (yes/no)	2,427 (86.9)/366 (13.1)
Self-rated health (SF.Total)	2.91 ± 1.23
Fitness self	2.26 ± 1.08
Worsen	3.15 ± 1.27
Fitness. same	2.18 ± 1.09
Illness (count)	3.39 ± 1.26

SF.Total, self-rated health total.

### 3.2 Correlations analysis among perceived built environment, physical activity, BMI, and self-rated health

Correlation analyses were conducted to examine the associations among perceived built environment, BMI, PA, and SRH (SF.Total). Overall, correlation coefficients were small (|*r*| ≤ 0.17).

#### 3.2.1 Perceived built environment and BMI

BMI showed very small correlations with individual perceived built environment items (|*r*| ≤ 0.08). At a two-sided α = 0.05, five items were nominally significant: Com.Env3 inversely, and Com.Env6, Com.Env15–17 is positively related to BMI. The composite perceived built environment score was non-significant (*r* = 0.037, *p* = 0.106).

#### 3.2.2 Perceived built environment and physical activity

Total metabolic equivalent (Total MET) displayed weak correlations with environmental items (|*r*| ≤ 0.12). Four items reached nominal significance (Com.Env1, Com.Env9 negative; Com.Env3, Com.Env13 positive). The composite environment score correlated weakly and non-significantly with physical activity (*r* = −0.028, *p* = 0.305). Following the FDR adjustment, only Com.Env13 retained significance.

### 3.3 Structure and modeling process of SEM

#### 3.3.1 Test of model assumptions

The data were suitable for factor analysis and structural equation modeling. The Kaiser–Meyer–Olkin value was 0.920, and Bartlett's test of sphericity was significant (χ^2^ = 34,933.87, df = 136, *p* < 0.001), indicating sufficient common variance among variables. Shapiro–Wilk and Mardia's tests revealed significant deviations from univariate and multivariate normality. Therefore, subsequent analyses were conducted using maximum likelihood estimation with robust (Huber–White) standard errors and Satorra–Bentler corrections. Detailed results are provided in Supplementary Table S7.

#### 3.3.2 Exploratory and confirmatory factor analysis (CFA)

An exploratory factor analysis was conducted to delineate the latent structure of the 17 perceived built environment function items. Factors were extracted using the minimum-residual (minres) method and rotated with oblimin to allow inter-factor correlations. A five-factor solution (CE1–CE5) was retained, cumulatively explaining 62.0% of the total variance; Most items loaded strongly on their respective factors (λ = 0.51–0.96), except for Com.Env13, which did not reach the 0.40 threshold. Confirmatory factor analysis further supported this structure, demonstrating acceptable construct validity (shown in Table 2).

**Table 2:** Factor loadings of the perceived built environment items.

**Variable**	**MR1**	**MR4**	**MR3**	**MR5**	**MR2**
Com.Env1			0.665		
Com.Env2			0.862		
Com.Env3			0.584		
Com.Env4			0.829		
Com.Env5	0.752				
Com.Env6	0.906				
Com.Env7	0.835				
Com.Env8	0.752				
Com.Env9	0.561				
Com.Env10				0.72	
Com.Env11				0.776	
Com.Env12					0.997
Com.Env13					
Com.Env14					0.51
Com.Env15		0.796			
Com.Env16		0.961			
Com.Env17		0.93			

Factors were extracted using the minimum-residual method and oblimin rotation. Loadings < 0.40 were suppressed. CE1–CE5 denote the five latent perceived built environment factors.

In summary, the data were suitable for factor analysis and SEM. Both univariate and multivariate tests indicated significant departures from normality, and robust estimation was therefore applied. Exploratory factor analysis identified five latent factors, which were further supported by confirmatory analysis. Detailed results of normality tests and factor loadings are provided in Supplementary Tables S7–S9.

### 3.4 Structural equation modeling (SEM)

#### 3.4.1 Model specification and identification

**Figure 1:**
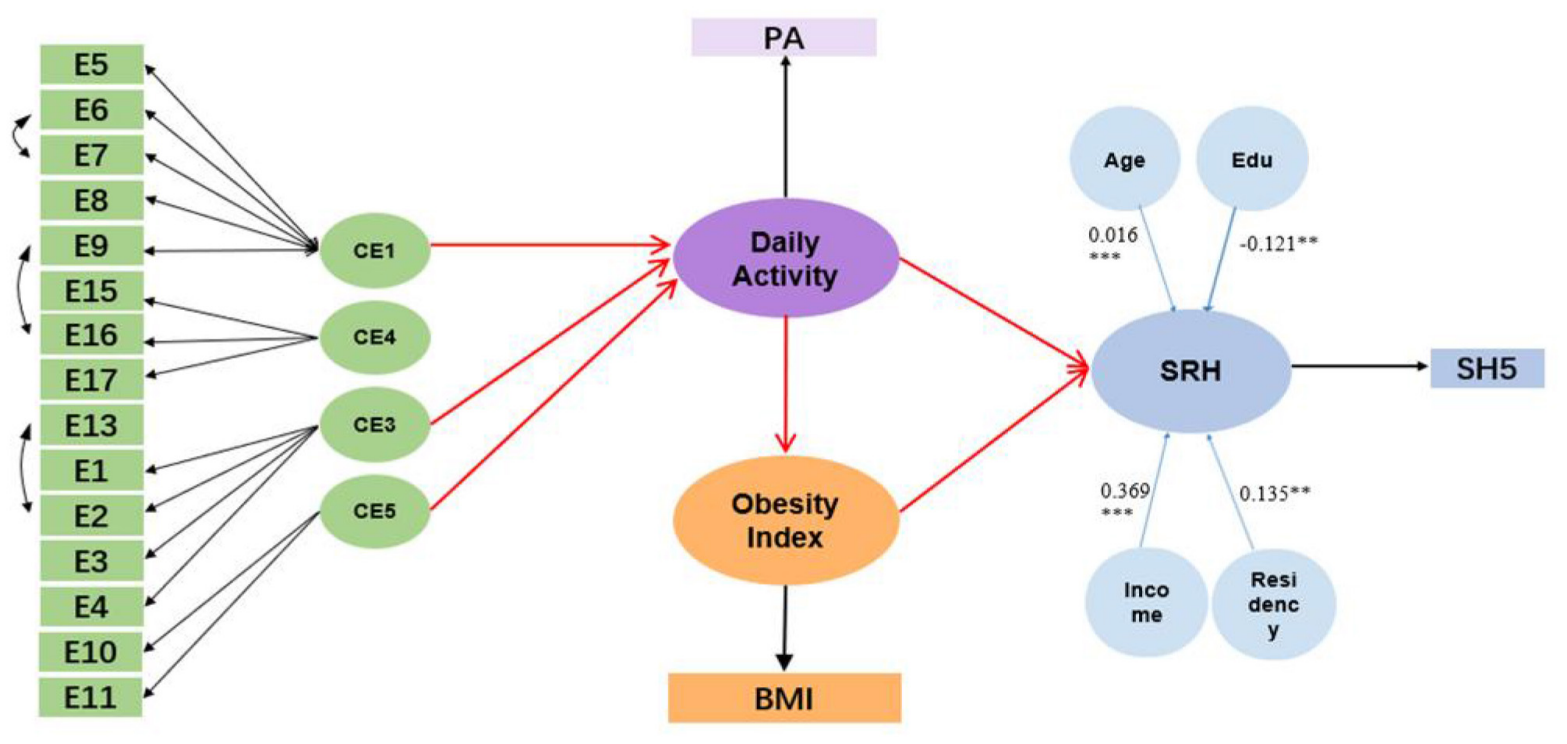
Structural equation model specification.

A structural equation model was specified with a measurement and a structural component. Five first-order latent perceived built environment factors (CE1–CE5) were defined by their respective item sets, while PA, BMI, and SRH were each modeled as latent constructs indicated by their observed measures. Residual variances were freely estimated, and latent factors were scaled by fixing one loading per factor to unity. The model was therefore overidentified and statistically estimable, satisfying the identification criteria for SEM (Figure 1).

#### 3.4.2 Model estimation and fit

The model demonstrated excellent overall fit after incorporating key covariates, which were age, gender, education, marital status, smoking, drinking, family economics and residency and single-indicator measurement errors. Fit indices were GFI = 0.999, AGFI = 0.998, CFI = 0.999, TLI = 0.999, RMSEA = 0.075, and RMR = 0.037 (Table 3).

**Table 3:** Summary of model fit indices and interpretation.

**Category**	**Index**	**Value**	**Common guidelines**	**Interpretation**
Absolute fit	GFI	0.999	≥0.90 acceptable. ≥0.95 excellent	Excellent
	AGFI	0.998	≥0.90 acceptable	Excellent
	RMSEA	0.075	≤ 0.05 close fit. 0.05–0.08 reasonable	Reasonable
	RMR	0.037	≤ 0.08 acceptable	Good (small residuals)
Incremental/ relative fit	CFI	0.999	≥0.95 excellent. ≥0.90 acceptable	Excellent
	TLI	0.999	≥0.95 excellent. ≥0.90 acceptable	Excellent
	NFI	0.999	≥0.90 acceptable	Excellent
	IFI	0.999	≥0.95 excellent. ≥0.90 acceptable	Excellent
Parsimony fit	PGFI	0.659	Higher = more parsimonious	Adequate
	PNFI	1.681	Higher = more parsimonious	Adequate

GFI, goodness-of-fit index; AGFI, adjusted GFI; RMSEA, root mean square error of approximation; RMR, root mean square residual; SRMR, standardized RMR; CFI, comparative fit index; TLI (NNFI), Tucker–Lewis index (non-normed fit index); NFI, normed fit index; IFI, incremental fit index; PGFI, parsimony GFI; PNFI, parsimony NFI.

#### 3.4.3 Measurement model

The measurement model indicated strong construct validity. All factors loading were significant (*p* < 0.001), with standardized loadings ranging from 0.76 to 0.94, confirming satisfactory convergent validity. Among the perceived built environment dimensions, CE4 (aesthetics) and CE5 (traffic conditions) demonstrated the strongest standardized loadings (λ = 0.89–0.92), while CE2 (living convenience) showed relatively moderate loadings (λ = 0.67–0.74).

Although two indicators (Com.Env6 and Com.Env15) exhibited lower loadings due to limited variance, their theoretical importance justified retention. The overall measurement model demonstrated stable psychometric properties and strong factorial validity (Table 4).

**Table 4:** Model estimates and statistics.

**Factor**	**Item**	**Est**	**SE**	***z***	***p***	**Std**	**CI lower**	**CI upper**
CE1	Com.Env6	182.866	20,542.651	0.009	0.993	1.000	−40,079.990	40,445.721
CE1	Com.Env5	8.328	2.038	4.087	0.000	0.993	4.334	12.322
CE1	Com.Env8	1.845	0.068	27.028	0.000	0.879	1.711	1.979
CE1	Com.Env7	1.777	0.064	27.674	0.000	0.871	1.651	1.903
CE1	Com.Env9	1.364	0.050	27.487	0.000	0.806	1.266	1.461
CE3	Com.Env4	1.854	0.089	20.760	0.000	0.880	1.679	2.029
CE3	Com.Env2	1.829	0.088	20.678	0.000	0.877	1.656	2.003
CE3	Com.Env1	1.819	0.102	17.885	0.000	0.876	1.619	2.018
CE3	Com.Env3	1.166	0.055	21.197	0.000	0.759	1.058	1.273
CE4	Com.Env15	89.201	3,817.845	0.023	0.000	1.000	−7,393.637	7,572.040
CE4	Com.Env16	2.820	0.167	16.875	0.000	0.943	2.493	3.148
CE5	Com.Env10	2.419	0.128	18.918	0.000	0.924	2.168	2.669
CE5	Com.Env11	1.824	0.076	24.151	0.000	0.877	1.676	1.972
Daily activity	Total_MET	1.000	0.000	NA	NA	1.000	1.000	1.000
Obesity index	BMI	1.000	0.000	NA	NA	1.000	1.000	1.000
SRH	SF.Total	1.000	0.000	NA	NA	1.000	1.000	1.000

#### 3.4.4 Structural model

The path analysis results of the structural equation model (Table 5) revealed that, following the adjustment for sociodemographic covariates, the direct and indirect effects of perceived built environment dimensions on SRH ceased to be statistically significant. Notable associations were identified mainly for the following covariates: age (β = 0.016, *p* < 0.001), household economic status (β = 0.369, *p* < 0.001), education (β = −0.121, *p* = 0.004), and residency (urban; β = 0.135, *p* = 0.006).

**Table 5:** Path relationship testing in structural equation modeling.

**Path**	**Estimate (β)**	**SE**	**C.R. (*t*-value)**	***p*-value**	**Significance**
**Primary hypotheses**					
Physical activity → SRH	−0.038	0.029	−1.342	0.180	No
BMI → SRH	0.028	0.028	0.996	0.319	No
CE1–CE5 → SRH/mediators	–	–	–	>0.05	No significant paths
**Socioeconomic and demographic covariates**					
Age → SRH	0.016	–	–	< 0.001	Yes
Education → SRH	−0.121	–	–	0.004	Yes
Household economic income → SRH	0.369	–	–	< 0.001	Yes
Residency → SRH	0.135	–	–	0.006	Yes

SRH, self-rated health; CE, perceived-built environments.

The analysis of direct, indirect, and total effects (Table 6) revealed that no significant pathways existed between perceived built environment and SRH via PA or BMI (all *p* > 0.05).

**Table 6:** Effect estimates and statistics.

**Effect**	**Est**	**SE**	***z***	***p***	**CI lower**	**CI upper**
dir_CE1_SRH	0.000	0.000	NA	NA	0.000	0.000
dir_CE3_SRH	0.000	0.000	NA	NA	0.000	0.000
dir_CE5_SRH	0.000	0.000	NA	NA	0.000	0.000
ind2_CE1_DA_OB_SRH	0.000	0.000	−0.135	0.893	0.000	0.000
ind2_CE3_DA_OB_SRH	0.000	0.000	−0.304	0.761	0.000	0.000
ind2_CE5_DA_OB_SRH	0.000	0.000	0.520	0.603	0.000	0.000
ind_CE1_DA_SRH	0.000	0.002	0.136	0.892	−0.004	0.005
ind_CE3_DA_SRH	0.001	0.002	0.323	0.747	−0.003	0.004
ind_CE5_DA_SRH	−0.002	0.003	−0.600	0.549	−0.007	0.004
ind_DA_OB_SRH	0.001	0.001	0.777	0.437	−0.001	0.003
ind_total_CE1_SRH	0.000	0.002	0.136	0.892	−0.004	0.005
ind_total_CE3_SRH	0.001	0.002	0.322	0.747	−0.003	0.004
ind_total_CE5_SRH	−0.002	0.003	−0.596	0.551	−0.007	0.004
te_CE1_on_Obesity	0.000	0.002	−0.137	0.891	−0.004	0.004
te_CE3_on_ Obesity	−0.001	0.002	−0.321	0.748	−0.004	0.003
te_CE5_on_ Obesity	0.001	0.002	0.630	0.529	−0.003	0.006
tot_CE1_on_SRH	0.000	0.002	0.136	0.892	−0.004	0.005
tot_CE3_on_SRH	0.001	0.002	0.322	0.747	−0.003	0.004
tot_CE5_on_SRH	−0.002	0.003	−0.596	0.551	−0.007	0.004
tot_DA_on_SRH	−0.383	0.029	−1.309	0.191	−0.094	0.019

SRH, self-rated health; CE, perceived built environment; OB, obesity; DA, daily activity.

## 4 Discussion

This study investigated the relationship between the perceived built environment and self-rated health (SRH) among community-dwelling middle-aged and older adults in western China, testing whether physical activity (PA) and BMI mediated this association while accounting for key socioeconomic and demographic factors. Although the parallel-mediation structural equation model (SEM) demonstrated excellent fit, the results diverged from traditional hypotheses. After adjustment for sociodemographic and economic covariates, neither the direct paths from perceived built environment domains to SRH nor the indirect paths via PA (β = −0.038, *p* = 0.180) or BMI (β = 0.028, *p* = 0.319) were statistically significant. Instead, SRH was mainly associated with household economic status, age, education, and urban residence.

It is well-documented that supportive built environments can benefit late-life health, often through high levels of physical activities and social support (Kloss et al., 2025). Extensive studies from Europe, North America, and Australia have identified that walkable, greener, and well-equipped neighborhoods promote better SRH, largely by facilitating PA and social engagement (Vos et al., 2020; Wang et al., 2024). However, the influence of the perceived built environment exhibits profound cultural, demographic, and geographical heterogeneity (Fan et al., 2023). The pathways observed in developed countries are highly contingent on specific urban planning paradigms that explicitly prioritize public physical activity spaces (Smith et al., 2017; Yang and Stone, 2024). In western China, characterized by rapid aging and relatively poor economic development, the magnitude of these environmental effects appears to be significantly attenuated (Vancampfort et al., 2017; Li et al., 2024).

The lack of a mediating impact through PA and BMI in our study is rooted in the unique spatial and cultural configuration of Chinese communities (Husu et al., 2024; Xu Y. et al., 2024; Kloss et al., 2025). In western China, the conceptualization of the perceived built environment differs fundamentally from the developed countries' models (Sun et al., 2024). For urban areas, high-density, gated communities are designed primarily to maximize residential capacity (Du et al., 2024). Given the dense population, these communities offer limited public spaces dedicated to physical activities for older adults (Gong et al., 2012). Conversely, over half of our sample resides in rural or town areas characterized by independent, self-built housing. In these vast, open settings, the modern concept of community exercise facilities is practically nonexistent (Zang et al., 2023). Consequently, for middle-aged and older adults in this region, a desirable perceived built environment is evaluated based on the convenience of daily life, such as access to transportation, markets, and healthcare, rather than the availability of physical activity facilities (Zajacova et al., 2014; Fan et al., 2023; Gao et al., 2024). This fundamental difference explains why the traditional perceived built environment to physical activity behavioral pathway failed to materialize in our cohort.

Furthermore, perceived built environmental features are inextricably linked to population backgrounds (Fischer et al., 2020; Blunden et al., 2023). Epidemiological evidence consistently highlights strong social gradients in SRH, where individuals with lower income or education are substantially more likely to report poor health (Liu et al., 2025). Our findings align with this pattern, suggesting that in economically underdeveloped and rapidly aging regions, socioeconomic position remains a far more proximal and decisive determinant of SRH (Xu T. et al., 2024). Once age, education, and household economic status were accounted for, the perceived built environment contributed little additional explanatory power. Rather than concluding that the perceived built environment is irrelevant, it is more accurate to state that its health-promoting effects are overshadowed, or diluted, by fundamental socioeconomic inequalities. In western China's settings like Nanchong, prioritizing the resolution of fundamental economic inequalities and guaranteeing access to healthcare supersedes the significance of minor differences in perceived built environmental characteristics.

This study has several limitations. First, self-rated health status is also influenced by genetic and clinical factors, which were not included. Second, the data were collected through a cross-sectional survey; it is not possible to use time-series methods to reduce the impact of bidirectional causality. Third, unobserved factors such as dietary patterns and healthcare access may confound the observed relationships. Future longitudinal or quasi-experimental studies that combine perceived and objective environmental data and include a wider range of social and behavioral variables are needed to clarify causal pathways in similar mixed urban, rural, aging contexts.

This study has several strengths. It is based on a relatively large sample of community-dwelling middle-aged and older adults in a less-studied western Chinese city and applies a parallel mediation within SEM to jointly examine direct and indirect pathways while accounting for socioeconomic and demographic factors.

## 5 Conclusion

In summary, while supportive perceived built environments are widely recognized for their health benefits, this study demonstrates that fundamental socioeconomic factors decisively outweigh environmental influences on self-rated health (SRH) in the rapidly aging and developing context of western China. Among community-dwelling middle-aged and older adults in Nanchong, SRH was predominantly determined by household economic status, educational level, age, and place of residency. Furthermore, neither physical activity nor body mass index mediated the relationship between the perceived built environment and SRH.

These findings indicate that in similar socioeconomically constrained and mixed urban-rural settings, environmental features alone are insufficient to overcome the health disparities driven by social gradients. Therefore, public health and urban planning strategies need to prioritize alleviating fundamental socioeconomic inequalities and ensuring equitable healthcare access.

## Data Availability

The raw data supporting the conclusions of this article will be made available by the authors, without undue reservation.
