# Supplementary material for: Socioeconomic factors outweigh perceived built environmental influences on self-rated health among middle-aged and older adults in western China
Source: Front Sociol. 2026 Mar 24;11:1784715. doi: 10.3389/fsoc.2026.1784715 (PMC13053311; doi:10.3389/fsoc.2026.1784715)
Supplement: Supplementary file 1 [file Supplementary_file_1.docx]

Supplementary Methods

**Figure S*1 Hypothesis**


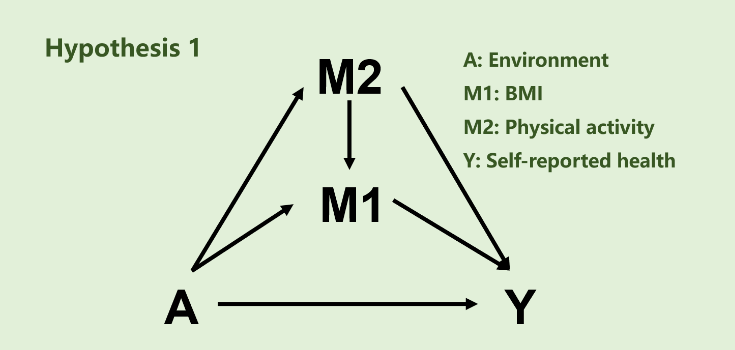


Figure S1, Hypothesised parallel-mediation model linking neighbourhood built environment (A) to perceived health (Y) through BMI (M1) and physical activity (M2).

H1 (Direct effect). The neighbourhood built environment is positively associated with perceived health in community-dwelling middle-aged and older adults.

H2 (A→M paths). The neighborhood built environment is associated with (a) higher physical activity and (b) healthier BMI.

H3 (M→Y paths). (a) Higher physical activity and (b) healthier BMI are each associated with better perceived health.

H4 (Parallel mediation). BMI and physical activity each mediate the association between the neighbourhood environment and perceived health (significant indirect effects via both mediators), constituting a parallel-mediation mechanism.

2.4 Measurements

**Table S*1 Variable Definitions and Coding**

| **Block** | | | **Variable** | | | **Coding** | **Notes for SEM** |
| --- | --- | --- | --- | --- | --- | --- | --- |
| Outcome | Perceived health | | | | SF-36 General Health (0–100, higher = better) | | Treated as continuous outcome |
| Exposure | Neighbourhood built environment | | | | Five domains (service convenience; road conditions; aesthetics; traffic conditions; public safety), harmonised so that higher = more supportive. | | Domain means (continuous); measured at individual level |
| Mediators | Body mass index (BMI) | | | | kg/m² (height / weight measured by trained staff) | | Continuous mediator |
|  | Physical activity | | | | Total MET-min/week (IPAQ-SF); log-transformed ln (MET+1) | | Continuous mediator  raw MET used only for descriptives |
| Covariates | | Age | | Years (continuous) | | | Exogenous covariate |
|  | | Sex | | 1=Male, 0=Female | | |  |
|  | | Education | | Ordinal (e.g., primary or below / secondary / tertiary) | | | Enter as dummy set if needed |
|  | | Marital status | | 1=non-single (married/cohabiting),  0=Single (never/sep/div/widowed) | | |  |
|  | | Household income | | Continuous (annual; local currency) or categorical (low/middle/high) | | | If categorical, use dummies |
|  | | Recent sickness/injury | | 1=No sickness/injury in last 2 weeks, 0=Yes | | | Health-status control |

**Table S*2. Classification of Study Locations and Environmental Characteristics**

| Administrative Classification (Chinese Term) | Functional Type (International Equivalent) | Typical Built Environment Characteristics (Planning Regulations) | Sampled Locations in This Study |
| --- | --- | --- | --- |
| Sub-district (Jiedao) | Urban Core | High Density  Multi-story residential apartments.  fully paved roads.  complete public utility coverage.  high access to commercial services and public transport. | Qinglian  Sub-district |
| Township (Zhen) | Peri-urban / Town | Medium Density  Mixed use of residential and small-scale commercial.  centralized living areas surrounded by semi-agricultural land; basic infrastructure present but less comprehensive than urban cores. | Zouma  Town |
| Village (Cun) | Rural Hinterland | Low Density  Detached housing or low-rise clusters.  dominant agricultural land use.  limited commercial services.  lower road connectivity and infrastructure standards. | Baihetang Village |

3.1 Results

**Table S*3. Baseline characteristics of the study population (N = 3,753)**

| Characteristic | Overall (N = 3,753) |
| --- | --- |
| **Gender** (n, %) |  |
| Male | 1,481 (39.5) |
| Female | 2,272 (60.5) |
| **Age** (years) | 50.13 ± 17.23 |
| **Ethnicity** (n, %) |  |
| Han | 3,726 (99.3) |
| Others | 27 (0.7) |
| **High Physical Activity** (n, %) |  |
| Yes | 727 (26.0) |
| No | 2,066 (74.0) |
| High Physical Activity Frequence  (days/week) | 3.73 ± 2.13 |
| High Physical Activity Hour  (hours/week) | 2.12 ± 2.86 |
| High Physical Activity Minute  (minutes/week) | 15.38 ± 14.75 |
| **Medium Physical Activity** (n, %) |  |
| Yes | 1,535 (55.0) |
| No | 1,258 (45.0) |
| Medium Physical Activity Frequence  (days/week) | 4.16 ± 2.23 |
| Medium Physical Activity Hour  (hours/week) | 1.70 ± 2.05 |
| Medium Physical Activity Minute  (minutes/week) | 14.67 ± 14.51 |
| **Walk Physical Activity** (n, %) |  |
| Yes | 2,427 (86.9) |
| No | 366 (13.1) |
| Walk Physical Activity Frequence  (days/week) | 5.40 ± 1.99 |
| Walk Physical Activity Hour  (hours/week) | 1.33 ± 1.73 |
| Walk Physical Activity Minute  (minutes/week) | 17.37 ± 14.94 |

**Table S*4 . Baseline Characteristics of the Study Population (N = 3,753)**

| **Characteristic** | **Scale Scores, mean ± SD** |
| --- | --- |
| Gender (n, %) | Overall (N = 3,753) |
| Male | 1,481 (39.5) |
| Female | 2,272 (60.5) |
| Age (years) | 50.13 ± 17.23 |
| FamFun1 | 2.93 ± 0.90 |
| FamFun2 | 1.74 ± 0.68 |
| FamFun3 | 2.80 ± 0.85 |
| FamFun4 | 1.97 ± 0.74 |
| FamFun5 | 2.75 ± 0.84 |
| FamFun6 | 1.90 ± 0.73 |
| FamFun7 | 3.04 ± 0.82 |
| FamFun8 | 2.02 ± 0.75 |
| FamFun9 | 2.95 ± 0.78 |
| FamFun10 | 1.88 ± 0.69 |
| FamFun11 | 3.12 ± 0.81 |
| FamFun12 | 1.77 ± 0.68 |
| Com.Env1 | 4.07 ± 1.06 |
| Com.Env2 | 3.89 ± 1.19 |
| Com.Env3 | 3.37 ± 1.39 |
| Com.Env4 | 3.75 ± 1.14 |
| Com.Env5 | 3.71 ± 1.04 |
| Com.Env6 | 3.73 ± 1.01 |
| Com.Env7 | 3.74 ± 1.06 |
| Com.Env8 | 3.88 ± 1.00 |
| Com.Env9 | 3.52 ± 1.13 |
| Com.Env10 | 3.64 ± 1.07 |
| Com.Env11 | 3.51 ± 1.07 |
| Com.Env12 | 2.51 ± 1.17 |
| Com.Env13 | 3.34 ± 1.05 |
| Com.Env14 | 2.75 ± 1.17 |
| Com.Env15 | 3.89 ± 0.98 |
| Com.Env16 | 4.00 ± 0.97 |
| Com.Env17 | 3.92 ± 0.97 |
| ADL1 | 1.04 ± 0.27 |
| ADL2 | 1.03 ± 0.23 |
| ADL3 | 1.06 ± 0.36 |
| ADL4 | 1.06 ± 0.34 |
| ADL5 | 1.06 ± 0.33 |
| ADL6 | 1.04 ± 0.30 |
| ADL7 | 1.18 ± 0.62 |
| ADL8 | 1.07 ± 0.39 |
| ADL9 | 1.09 ± 0.42 |
| ADL10 | 1.10 ± 0.45 |
| ADL11 | 1.08 ± 0.42 |
| ADL12 | 1.14 ± 0.54 |
| ADL13 | 1.04 ± 0.30 |
| ADL14 | 1.09 ± 0.39 |
| Sleep.disorder1 | 1.89 ± 1.02 |
| Sleep.disorder2 | 1.88 ± 1.03 |
| Sleep.disorder3 | 1.85 ± 1.00 |
| Sleep.disorder4 | 2.36 ± 1.12 |
| Sleep.disorder5 | 1.88 ± 0.97 |
| Sleep.disorder6 | 1.86 ± 1.00 |
| Sleep.disorder7 | 1.91 ± 1.03 |
|  |  |

Note: FamFun means Family Functions, ADL means Activity of Daily Life, Com.Env means the built environment.

3.2 Correlation analysis between exposures, mediators, and outcomes

3.2.1 Exposure – M1 – BMI


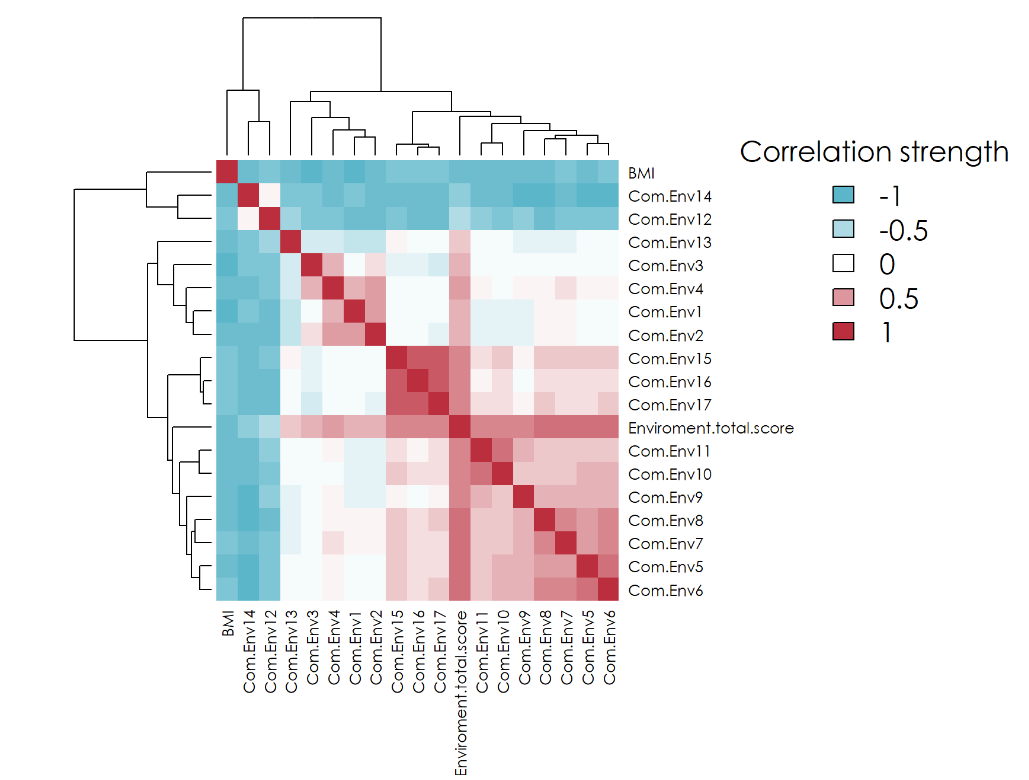


**Correlation analysis and hierarchical-clustering heatmap**

We examined pairwise correlations between body-mass index (BMI) and 18 community-built environment variables. Effect sizes were uniformly small (|r| ≤ 0.08). At a two-sided α of 0.05, five-item–BMI correlations were nominally significant (Com.Env3 inversely; Com.Env6, Com.Env15, Com.Env16, and Com.Env17 positively). The composite Environment.total.score showed a very small, non-significant association with BMI (r = 0.037; P = 0.106).

The hierarchical-clustering heatmap demonstrates two coherent modules. First, a dense block comprising Environment.total.score with Com.Env5–Com.Env11 and Com.Env15–Com.Env17 exhibits strong positive intercorrelations. Second, a sparser BMI-anchored cluster—including Com.Env12–Com.Env14 and neighbouring items—shows weak negative to near-null relations with the other variables. BMI occupies a peripheral position in the network, which accords with the small pairwise r values observed in the table. Data is Pearson’s correlation coefficients with two-sided P values and no multiplicity adjustment.

**TableS*5. Pairwise correlations between BMI and community-built environment variables**

| **Variable 1** | **Variable 2** | **r** | **P value** |
| --- | --- | --- | --- |
| Enviroment.total.score | BMI | 0.037 | 0.106 |
| Com.Env1 | BMI | 0.040 | 0.085 |
| Com.Env2 | BMI | 0.007 | 0.749 |
| Com.Env3 | BMI | -0.056 | 0.014 |
| Com.Env4 | BMI | 0.016 | 0.473 |
| Com.Env5 | BMI | 0.013 | 0.581 |
| Com.Env6 | BMI | 0.061 | 0.007 |
| Com.Env7 | BMI | 0.039 | 0.085 |
| Com.Env8 | BMI | 0.033 | 0.153 |
| Com.Env9 | BMI | 0.037 | 0.108 |
| Com.Env10 | BMI | 0.034 | 0.134 |
| Com.Env11 | BMI | 0.034 | 0.143 |
| Com.Env12 | BMI | -0.008 | 0.738 |
| Com.Env13 | BMI | -0.020 | 0.380 |
| Com.Env14 | BMI | -0.018 | 0.435 |
| Com.Env15 | BMI | 0.080 | 0.000 |
| Com.Env16 | BMI | 0.050 | 0.031 |
| Com.Env17 | BMI | 0.077 | 0.001 |

3.2.2 Exposure – M2 – Physical Activity


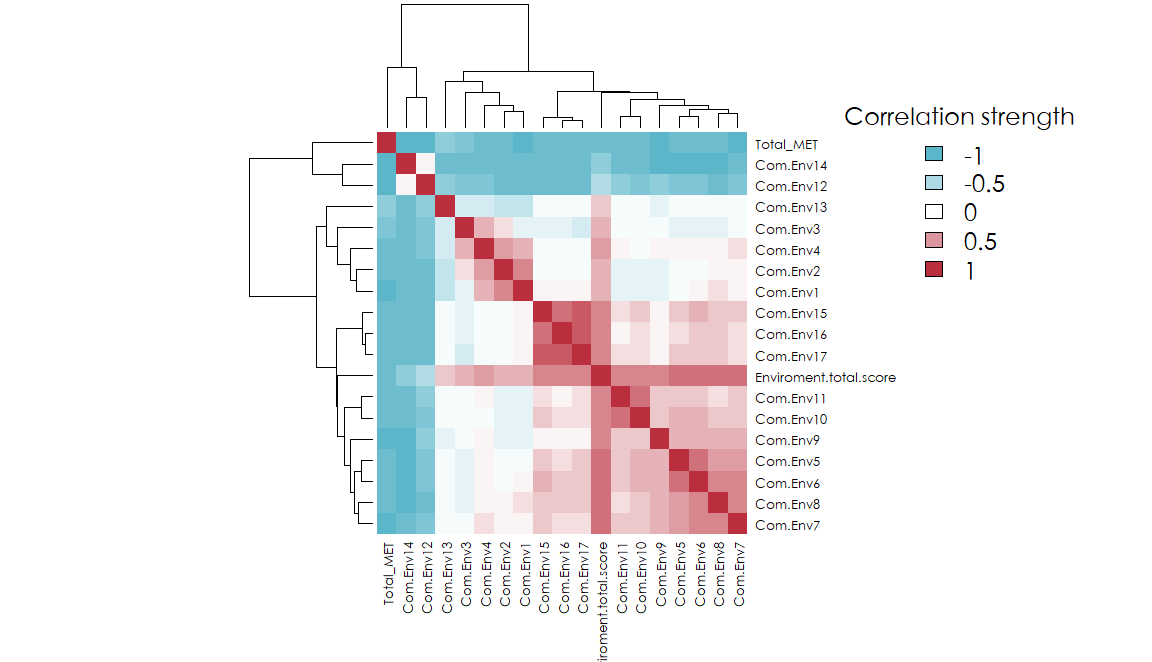


**Correlation analysis and hierarchical-clustering heatmap (Total MET)**

We assessed pairwise correlations between total metabolic equivalent of task (Total MET) and 18 community‑built environment variables. Effect sizes were small (|r| ≤ 0.121). At a nominal two‑sided α of 0.05, four item–Total MET correlations reached significance (Com.Env1 and Com.Env9 inversely; Com.Env3 and Com.Env13 positively). The composite Enviroment.total.score had a very small, non‑significant correlation with Total MET (r = −0.028; P = 0.305).

After controlling the false discovery rate at 5% (Benjamini–Hochberg), only Com.Env13 remained significant.

The hierarchical‑clustering heatmap shows two clear modules. First, a tightly inter‑correlated block—anchored by Enviroment.total.score and comprising Com.Env5–Com.Env11 and Com.Env15–Com.Env17—exhibits predominantly positive within‑block correlations (warmer tiles). Second, a sparser cluster anchored by Total MET and including Com.Env12–Com.Env14 shows weak negative to near‑null associations with the remaining variables. Total MET occupies a peripheral position in the network, consistent with the small pairwise correlations.

**Table S*6 Pairwise correlations between Total MET and perceived built environment**

| **Variable 1** | **Variable 2** | **r** | **P value** |
| --- | --- | --- | --- |
| Enviroment.total.score | Total_MET | -0.028 | 0.305 |
| Com.Env1 | Total_MET | -0.071 | 0.009 |
| Com.Env2 | Total_MET | -0.017 | 0.540 |
| Com.Env3 | Total_MET | 0.074 | 0.006 |
| Com.Env4 | Total_MET | -0.026 | 0.335 |
| Com.Env5 | Total_MET | -0.011 | 0.698 |
| Com.Env6 | Total_MET | -0.038 | 0.164 |
| Com.Env7 | Total_MET | -0.040 | 0.141 |
| Com.Env8 | Total_MET | -0.034 | 0.208 |
| Com.Env9 | Total_MET | -0.056 | 0.039 |
| Com.Env10 | Total_MET | -0.015 | 0.572 |
| Com.Env11 | Total_MET | -0.013 | 0.624 |
| Com.Env12 | Total_MET | -0.036 | 0.182 |
| Com.Env13 | Total_MET | 0.121 | 0.000 |
| Com.Env14 | Total_MET | -0.036 | 0.180 |
| Com.Env15 | Total_MET | -0.007 | 0.784 |
| Com.Env16 | Total_MET | 0.026 | 0.344 |
| Com.Env17 | Total_MET | -0.009 | 0.729 |

3.2.3 Correlation between M1 and M2


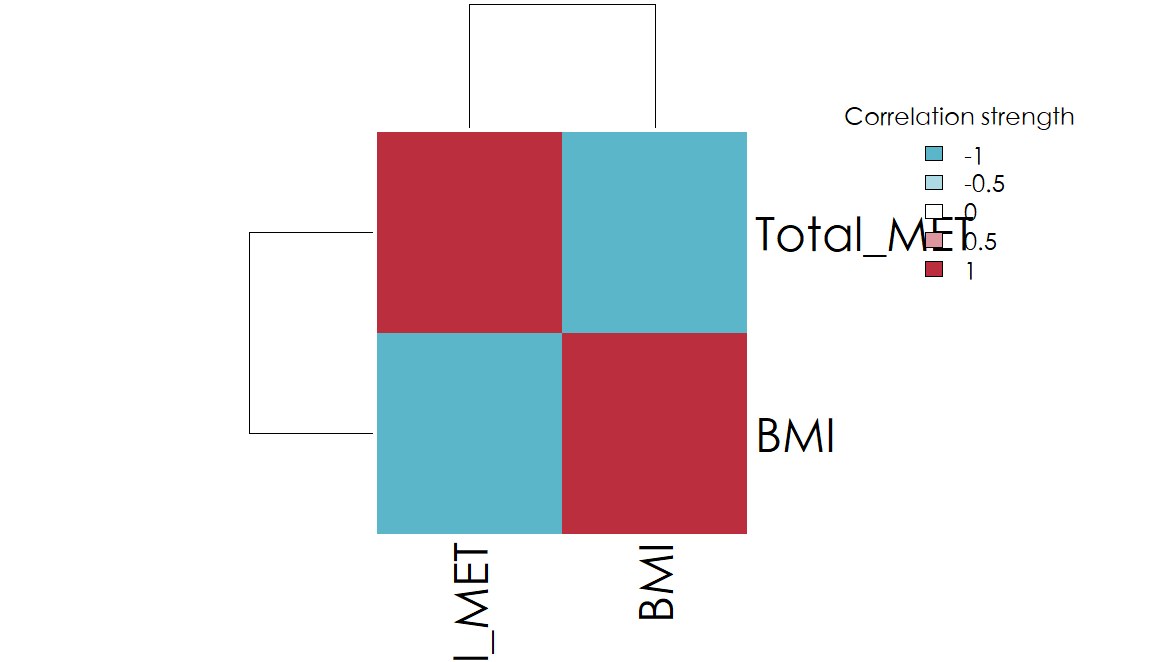
 Row column cor p value

Total_MET BMI 0.301 0.056

3.2.4 Exposure – Y – Self-rated Health


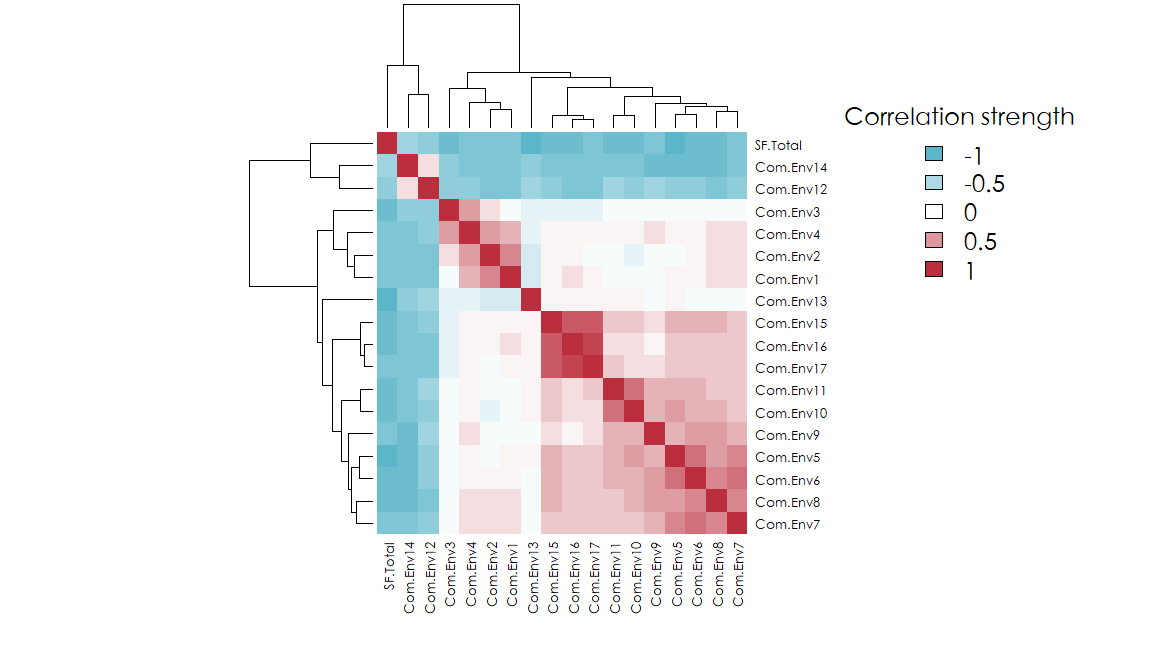


**Correlation analysis and hierarchical-clustering heatmap (SF.Total)**

We analysed pairwise correlations between the SF.Total score and 17 community-built environment variables. Effect sizes ranged from −0.167 to 0.122, indicating generally small associations. At a two-sided α of 0.05, significant inverse correlations were observed for Com.Env3, Com.Env5, Com.Env6, Com.Env7, Com.Env8, Com.Env10, Com.Env11, Com.Env15, Com.Env16, and Com.Env17; positive correlations were observed for Com.Env12 and Com.Env14. Among these, Com.Env13 showed the largest magnitude (r = −0.167; P < 0.001).

After Benjamini–Hochberg control of the false discovery rate at 5%, the following items remained significant: Com.Env3, Com.Env5, Com.Env6, Com.Env7, Com.Env8, Com.Env10, Com.Env11, Com.Env12, Com.Env13, Com.Env14, Com.Env15, Com.Env16, Com.Env17.

The hierarchical-clustering heatmap reveals two coherent structures. SF.Total clusters on the fringe together with Com.Env14 and Com.Env12, reflecting their positive relations. In contrast, a dense block composed of Com.Env5–Com.Env11 and Com.Env15–Com.Env17 shows strong positive within-block correlations. Cross-block relations are weakly negative or near null, consistent with SF.Total’s predominantly inverse correlations with many environmental items.

**Table S*7. Pairwise correlations between SF.Total and perceived built environment**

| **Variable 1** | **Variable 2** | **r** | **P value** |
| --- | --- | --- | --- |
| SF.Total | Com.Env1 | -0.020 | 0.463 |
| SF.Total | Com.Env2 | -0.043 | 0.108 |
| SF.Total | Com.Env3 | -0.086 | 0.001 |
| SF.Total | Com.Env4 | -0.026 | 0.343 |
| SF.Total | Com.Env5 | -0.133 | 0.000 |
| SF.Total | Com.Env6 | -0.072 | 0.008 |
| SF.Total | Com.Env7 | -0.064 | 0.018 |
| SF.Total | Com.Env8 | -0.086 | 0.001 |
| SF.Total | Com.Env9 | -0.031 | 0.249 |
| SF.Total | Com.Env10 | -0.107 | 0.000 |
| SF.Total | Com.Env11 | -0.082 | 0.002 |
| SF.Total | Com.Env12 | 0.115 | 0.000 |
| SF.Total | Com.Env13 | -0.167 | 0.000 |
| SF.Total | Com.Env14 | 0.122 | 0.000 |
| SF.Total | Com.Env15 | -0.104 | 0.000 |
| SF.Total | Com.Env16 | -0.095 | 0.000 |
| SF.Total | Com.Env17 | -0.077 | 0.004 |

Note: SF.Total means Self-rated Health total scale

**Table S*8. Pearson Correlation Matrix of Variables**

| **Variables** | **SRH** | **Com. Env** | **PA** | **BMI** | **Age** | **Education** | **Income†** | **Residency** |
| --- | --- | --- | --- | --- | --- | --- | --- | --- |
| SRH | 1 |  |  |  |  |  |  |  |
| Com. Env | 0.172** | 1 |  |  |  |  |  |  |
| PA | 0.008 | -0.036 | 1 |  |  |  |  |  |
| BMI | 0.026 | -0.005 | 0.051 | 1 |  |  |  |  |
| Age | -0.225** | 0.03 | -0.046 | -0.102** | 1 |  |  |  |
| Education | 0.268** | 0.05 | 0.070* | 0.023 | -0.612** | 1 |  |  |
| Income† | -0.260** | -0.114** | -0.007 | -0.094** | 0.104** | -0.203** | 1 |  |
| Residency | -0.032 | 0.270** | 0.02 | 0.012 | 0.118** | -0.080* | 0.002 | 1 |

Note: * p < 0.05, ** p < 0.01. † Income coded as 1=Good to 5=Poor, thus negative correlation indicates higher income relates to better health. Correlation analysis was performed on valid cases with complete data for all socioeconomic variables.

- 1. Structure and modeling procession of SEM

3.3.1 Test of model assumptions

To evaluate the suitability of the data for factor analysis and structural equation modeling, several assumptions were tested, including measures of sampling adequacy, variable intercorrelations, univariate and multivariate normality. The assumption of sampling adequacy was tested using the Kaiser–Meyer–Olkin (KMO) measure and Bartlett’s test of sphericity. Results demonstrated a KMO value of 0.920 and a significant Bartlett’s ($x^{2}$34,933.87, df = 136, P < 0.001), indicating sufficient common variance and necessary intercorrelations among the seventeen Community Environment Function variables for factor analysis. Then, the assumption of univariate normality was evaluated using the Shapiro–Wilk test, which revealed significant deviations from normality across all observed variables (P < 0.05 for each item). The assumption of multivariate normality was examined with Mardia’s test, and both skewness (11,290.99, P < 0.001) and kurtosis (170.00, P < 0.001) were significant, confirming a violation of the multivariate normal distribution.

To address these violations, subsequent analyses were conducted using maximum likelihood estimation with robust (Huber–White) standard errors and Satorra–Bentler scaled test statistics. Correlation analyses further revealed substantial interrelationships among the observed variables, supporting their inclusion in latent structure modeling.

**Table S*9. Shapiro–Wilk Test for Univariate Normality**

| **Variable** | **W** | **P-value** | **Normality** |
| --- | --- | --- | --- |
| Com.Env1 | 0.801 | <0.001 | No |
| Com.Env2 | 0.830 | <0.001 | No |
| Com.Env3 | 0.875 | <0.001 | No |
| Com.Env4 | 0.863 | <0.001 | No |
| Com.Env5 | 0.877 | <0.001 | No |
| Com.Env6 | 0.875 | <0.001 | No |
| Com.Env7 | 0.865 | <0.001 | No |
| Com.Env8 | 0.850 | <0.001 | No |
| Com.Env9 | 0.891 | <0.001 | No |
| Com.Env10 | 0.881 | <0.001 | No |
| Com.Env11 | 0.890 | <0.001 | No |
| Com.Env12 | 0.894 | <0.001 | No |
| Com.Env13 | 0.903 | <0.001 | No |
| Com.Env14 | 0.911 | <0.001 | No |
| Com.Env15 | 0.853 | <0.001 | No |
| Com.Env16 | 0.832 | <0.001 | No |
| Com.Env17 | 0.848 | <0.001 | No |

W = Shapiro–Wilk statistic. All items significantly deviated from normality (P < 0.001).

3.3.2 CFA

An exploratory factor analysis was conducted to delineate the latent structure of the 17 Community Environment Function items. Factors were extracted using the minimum-residual (minres) method and rotated with oblimin to allow inter-factor correlations. A five-factor solution was retained; loadings < 0.40 were suppressed for interpretability. The rotated pattern showed that Com.Env1–Com.Env4 loaded on MR3 (0.584–0.862), Com.Env5–Com.Env9 on MR1 (0.561–0.906), Com.Env10–Com.Env11 on MR5 (0.720, 0.776), Com.Env15–Com.Env17 on MR4 (0.796–0.961), and Com.Env12–Com.Env14 on MR2 (0.510–0.997), whereas Com.Env13 did not reach the 0.40 threshold. Factor sums of squared loadings were 3.080 (MR1), 2.556 (MR4), 2.251 (MR3), 1.381 (MR5), and 1.265 (MR2), accounting for 18.1%, 15.0%, 13.2%, 8.1%, and 7.4% of variance, respectively; cumulatively, the five factors explained 62.0% of the total variance.

**Table S*10. Factor Loadings of the Community Environment Function Items**

| **Variable** | **MR1** | **MR4** | **MR3** | **MR5** | **MR2** |
| --- | --- | --- | --- | --- | --- |
| Com.Env1 |  |  | 0.665 |  |  |
| Com.Env2 |  |  | 0.862 |  |  |
| Com.Env3 |  |  | 0.584 |  |  |
| Com.Env4 |  |  | 0.829 |  |  |
| Com.Env5 | 0.752 |  |  |  |  |
| Com.Env6 | 0.906 |  |  |  |  |
| Com.Env7 | 0.835 |  |  |  |  |
| Com.Env8 | 0.752 |  |  |  |  |
| Com.Env9 | 0.561 |  |  |  |  |
| Com.Env10 |  |  |  | 0.72 |  |
| Com.Env11 |  |  |  | 0.776 |  |
| Com.Env12 |  |  |  |  | 0.997 |
| Com.Env13 |  |  |  |  |  |
| Com.Env14 |  |  |  |  | 0.51 |
| Com.Env15 |  | 0.796 |  |  |  |
| Com.Env16 |  | 0.961 |  |  |  |
| Com.Env17 |  | 0.93 |  |  |  |

Factors were extracted using the minimum-residual method and oblimin rotation. Loadings < 0.40 were suppressed. CE1–CE5 denote the five latent community environment factors.

**Table S*11 Factor Summary Statistics**

| **Factor** | **SS Loadings** | **Proportion Var** | **Cumulative Var** |
| --- | --- | --- | --- |
| MR1 | 3.08 | 0.181 | 0.181 |
| MR4 | 2.556 | 0.15 | 0.332 |
| MR3 | 2.251 | 0.132 | 0.464 |
| MR5 | 1.381 | 0.081 | 0.545 |
| MR2 | 1.265 | 0.074 | 0.62 |

Note: SS Loadings = sum of squared loadings. Proportion Var = proportion of variance explained. Cumulative Var = cumulative variance explained.

3.4.3 Structural model

The measurement model demonstrated strong and statistically significant relationships between the latent constructions and their observed indicators. All factor loadings were significant at *P* < 0.001, supporting the construct validity of the measured variables. For the construct CE1, all five items exhibited high factor loadings, ranging from 0.777 (Com.Env9) to 0.880 (Com.Env6), accounting for 60.4% to 77.4% of the variance (R²). Similarly, CE4 was well represented by its three indicators, with loadings between 0.891 and 0.947, explaining between 79.3% and 89.7% of the variance.CE3 was measured by four items, with loadings from 0.653 (Com.Env3) to 0.852 (Com.Env4), and variance explained ranging from 42.7% to 72.6%. CE5 showed very high loadings of 0.881 and 0.920 for its two items, Com.Env11 and Com.Env10, respectively, with R² values of 77.6% and 84.6%.CE2 displayed solid loadings of 0.737 (Com.Env12) and 0.674 (Com.Env14), explaining 54.3% and 45.4% of the variance, respectively.

The Self.report.Health construct was measured by four indicators, with two negative and two positive loadings. Fitness.self showed the strongest negative relationship (λ = -0.830, R² = 0.688), followed by Fitness.same (λ = -0.804, R² = 0.646). The positive indicators Illness and Worsen had loadings of 0.483 and 0.489, explaining 23.4% and 24.0% of the variance, respectively.

Overall, the measurement model indicated that the latent variables were adequately represented by their respective observed indicators, with most items demonstrating moderate to strong loadings and acceptable levels of explained variance.

**Table S*12. Factor Loadings and *R²* of the Measurement Model**

| **Latent Variable** | **Observed Variable** | **Coefficient** | ***Z*** | ***P*** | ***R²*** |
| --- | --- | --- | --- | --- | --- |
| **CE1** | Com.Env6 | 0.880 | 73.503 | <0.001 | 0.774 |
|  | Com.Env7 | 0.868 | 70.279 | <0.001 | 0.754 |
|  | Com.Env5 | 0.843 | 59.885 | <0.001 | 0.710 |
|  | Com.Env8 | 0.861 | 68.147 | <0.001 | 0.741 |
|  | Com.Env9 | 0.777 | 46.961 | <0.001 | 0.604 |
| **CE4** | Com.Env16 | 0.891 | 45.535 | <0.001 | 0.793 |
|  | Com.Env17 | 0.915 | 47.010 | <0.001 | 0.838 |
|  | Com.Env15 | 0.947 | 91.146 | <0.001 | 0.897 |
| **CE3** | Com.Env2 | 0.845 | 57.386 | <0.001 | 0.715 |
|  | Com.Env4 | 0.852 | 55.041 | <0.001 | 0.726 |
|  | Com.Env1 | 0.792 | 38.188 | <0.001 | 0.627 |
|  | Com.Env3 | 0.653 | 31.158 | <0.001 | 0.427 |
| **CE5** | Com.Env11 | 0.881 | 60.418 | <0.001 | 0.776 |
|  | Com.Env10 | 0.920 | 91.619 | <0.001 | 0.846 |
| **CE2** | Com.Env12 | 0.737 | 10.920 | <0.001 | 0.543 |
|  | Com.Env14 | 0.674 | 10.779 | <0.001 | 0.454 |
| **SRH** | Illness | 0.483 | 14.822 | <0.001 | 0.234 |
|  | Fitness.same | -0.804 | -30.698 | <0.001 | 0.646 |
|  | Worsen | 0.489 | 15.753 | <0.001 | 0.240 |
|  | Fitness.self | -0.830 | -42.241 | <0.001 | 0.688 |
